# The Baseline Structure of the Enteric Nervous System and Its Role in Parkinson’s Disease

**DOI:** 10.3390/life11080732

**Published:** 2021-07-22

**Authors:** Gianfranco Natale, Larisa Ryskalin, Gabriele Morucci, Gloria Lazzeri, Alessandro Frati, Francesco Fornai

**Affiliations:** 1 Department of Translational Research and New Technologies in Medicine and Surgery, University of Pisa, 56126 Pisa, Italy; larisa.ryskalin@unipi.it (L.R.); gabriele.morucci@unipi.it (G.M.); gloria.lazzeri@unipi.it (G.L.); francesco.fornai@unipi.it (F.F.); 2 Museum of Human Anatomy “Filippo Civinini”, University of Pisa, 56126 Pisa, Italy; 3 Neurosurgery Division, Human Neurosciences Department, Sapienza University of Rome, 00135 Rome, Italy; alessandro.frati@uniroma1.it; 4 Istituto di Ricovero e Cura a Carattere Scientifico (I.R.C.C.S.) Neuromed, 86077 Pozzilli, Italy

**Keywords:** gastrointestinal tract, enteric nervous system, microbiota–gut–brain axis, Parkinson’s disease, neurodegeneration, α-synuclein, dopamine

## Abstract

The gastrointestinal (GI) tract is provided with a peculiar nervous network, known as the enteric nervous system (ENS), which is dedicated to the fine control of digestive functions. This forms a complex network, which includes several types of neurons, as well as glial cells. Despite extensive studies, a comprehensive classification of these neurons is still lacking. The complexity of ENS is magnified by a multiple control of the central nervous system, and bidirectional communication between various central nervous areas and the gut occurs. This lends substance to the complexity of the microbiota–gut–brain axis, which represents the network governing homeostasis through nervous, endocrine, immune, and metabolic pathways. The present manuscript is dedicated to identifying various neuronal cytotypes belonging to ENS in baseline conditions. The second part of the study provides evidence on how these very same neurons are altered during Parkinson’s disease. In fact, although being defined as a movement disorder, Parkinson’s disease features a number of degenerative alterations, which often anticipate motor symptoms. Among these, the GI tract is often involved, and for this reason, it is important to assess its normal and pathological structure. A deeper knowledge of the ENS is expected to improve the understanding of diagnosis and treatment of Parkinson’s disease.

## 1. Introduction

The gastrointestinal (GI) tract is a long tubular structure whose wall is deputed to important and complex functions in terms of motor activity, absorption, and secretion. For this sophisticated activity, the GI tract is provided with a peculiar nervous network, known as the enteric nervous system (ENS), deputed to the fine control and regulation of digestive functions. The ENS derives from pre-enteric vagal (hindbrain) as well as sacral neural crest cells and includes efferent and afferent neurons and interneurons, making this system capable of carrying reflexes in the absence of inputs from the central nervous system (CNS). Then, this network is relatively independent of the classic sympathetic and parasympathetic innervations, allowing the onset of modulated local responses [1,2,3].

Furthermore, this large variety of nervous cells is not a simple collection of relay ganglia but a well-organized and integrated network of plexuses. For this reason, the particular anatomical arrangement and functional properties of the ENS led to the definition of “second brain” [4]. In this respect, the GI tract was also defined as a “neurological organ” [5]. Nevertheless, when comparing the nervous system across the animal kingdom, it can be noted that the ENS occurs in all animal species, including hydra, echinoderms, and hemichordates that do not have a CNS. Then, paradoxically, it can be concluded that the ENS arose before and independently of the CNS and can be regarded as the real “first brain” [6]. In addition to this, more recently, the presence of the intestinal microbiota, with different strains of bacteria and other microorganisms, has been recognized as a further challenge for the intestinal wall and its cytotypes, leading to intriguing interactions among nervous, epithelial, hormonal, and immune responses [7].

**Figure 1:**
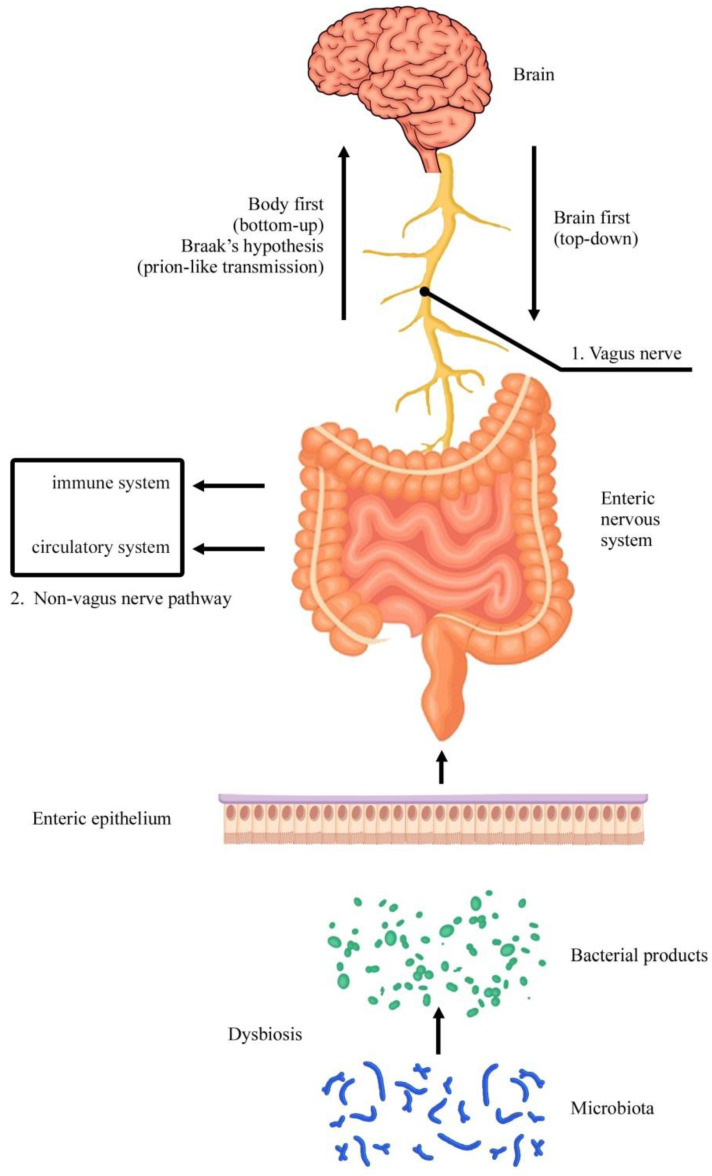
Microbiota–gut–brain axis. Two subtypes of Parkinson’s disease patients have been recognized: a brain-first (top-down) type, where the hallmark of this pathology (α-synuclein) initially appears in the brain with secondary spreading to the peripheral autonomic nervous system; a body-first (bottom-up) type, where the pathology originates in the enteric or peripheral autonomic nervous system and then spreads to the brain. In this case, according to Braak’s hypothesis, neurodegenerative diseases, such as Parkinson’s disease, can recognize a peripheral origin when putative pathogens enter the mucosa of the GI tract, inducing misfolding and aggregation of α-synuclein in the ENS, and finally spreading retrogradely to CNS via vagal preganglionic fibers, up to the dorsal motor nucleus of this nerve and finally other central nervous structures. A prion-like mechanism has also been proposed for misfolded proteins propagation, and different routes have been hypothesized to account for this cell-to-cell spreading, including vagal (1) and non-vagal (immune and circulatory systems) (2) pathways.

Of course, what occurs in the GI tract is also monitored and regulated by different structures of the CNS, with relevant biochemical signaling deputed to govern homeostasis through nervous, endocrine, immune, and metabolic pathways. An important role in this communication system is represented by the hypothalamus-pituitary gland-adrenal gland axis. More in-depth, to broadly define this articulated relationship, a gut–brain axis, or better microbiota–gut–brain axis, has been bidirectionally described. It is now well ascertained that there is a reciprocal communication between the brain and GI tract [8] (Figure 1).

Considering this complexity, aging and severe inflammatory (irritable bowel disease and Crohn’s disease) and neurodegenerative pathological conditions must be taken into consideration in the GI tract. According to the classic Braak’s hypothesis [9], neurodegenerative diseases, in particular Parkinson’s disease, can recognize a peripheral origin when putative pathogens enter the mucosa of the GI tract, inducing misfolding and aggregation of the hallmark of this pathology, α-synuclein, in specific neuron subtypes of the ENS, and finally spreading retrogradely to CNS via vagal preganglionic fibers, up to the dorsal motor nucleus of this nerve and finally other central nervous structures [10,11,12,13,14]. More specifically, a prion-like mechanism has also been proposed for misfolded proteins propagation, including α-synuclein, and different mechanisms have been hypothesized to account for this cell-to-cell spreading during neurodegeneration. Then, both vagal and non-vagal (immune and circulatory system) pathways seem to be involved, also including exosomes, tunneling nanotubes, and trogocytosis [15,16,17,18,19,20,21,22] (Figure 1).

In particular, two main subtypes of Parkinson’s disease patients have been recently recognized: a brain-first (top-down) type, where α-synuclein pathology initially arises in the brain with secondary spreading to the peripheral autonomic nervous system; and a body-first (bottom-up) type, where the pathology originates in the enteric or peripheral autonomic nervous system and then spreads to the brain [23,24,25]. Animal models of Parkinson’s disease also showed that α-synuclein may have the ability to traffic in a bidirectional manner along the gut–brain axis and through the blood–brain barrier, and that α-synuclein pathology in the CNS may impact upon the ENS and gut health with the involvement of gut microbiome [26] (Figure 1).

The present article aims to focus the attention on the ENS cell population in an attempt to examine which cytotypes are particularly involved in neurodegenerative processes, with special consideration to Parkinson’s disease. As outlined before, in spite of its typical neurological manifestations, Parkinson’s disease is now regarded as a systemic pathology, and several autonomic alterations have been described in peripheral organs as prodromal symptoms and markers [27]. The GI tract is widely affected, hence the importance of assessing the early alterations occurring in the ENS and interpreting their role in the pathogenesis of Parkinson’s disease. This body of knowledge is important for the implications in pathophysiology, diagnosis, and development of therapeutic strategies.

## 2. Methods

A systematic review of the literature was conducted using PubMed, Scopus, and Web of Science bibliographic databases, searching and crossing the following main keywords: gastrointestinal tract; enteric nervous system; enteric glia; microbiota–gut–brain axis; gut–brain axis; vagus nerve; enteric dysbiosis; Parkinson’s disease; neurodegenerative disease; α-synuclein; dopamine; and prionoids. All extracted data were from both review and original selected articles.

The article has been organized into two main parts.

(1) The first one concerns the neuroanatomy of ENS. In detail, a historical background was provided from the recognition of an autonomous nervous network in the GI tract up to the employment of a vast number of classification methods based on different criteria (morphological, electrophysiological, biochemical, retrograde tracing, transcriptome analysis, etc.), leading to the identification of several cytotypes;
(2) The second part focuses the attention on the ENS cell population to examine which cytotypes are particularly involved in Parkinson’s disease. Original articles were chronologically examined. The involvement of the GI tract in this neurodegenerative pathology was reported in humans as well as in animals. In particular, different models of experimental Parkinsonism were examined and discussed. Vagal and extra-vagal mechanisms were described. The recent involvement of microbiota was considered.

## 3. Organization of the ENS

Although the idea of an ENS independent from CNS was proposed by Langley in 1921 [28], this observation was seriously considered only in the sixties. In fact, in 1964, Burnstock wondered whether there were intrinsic nerves distinct from sympathetic ones, and his studies finally contributed to abandoning the previous idea that the neurons within the gut wall were merely post-ganglionic neurons of parasympathetic pathways [29]. The discovery of non-adrenergic, non-cholinergic (NANC) neurons (metasympathetic nervous system) also reinforced the novel view of the presence in the GI tract of intrinsic neurons organized into two principal plexuses, myenteric and submucosal, which are able to generate local reflexes.

**Figure 2:**
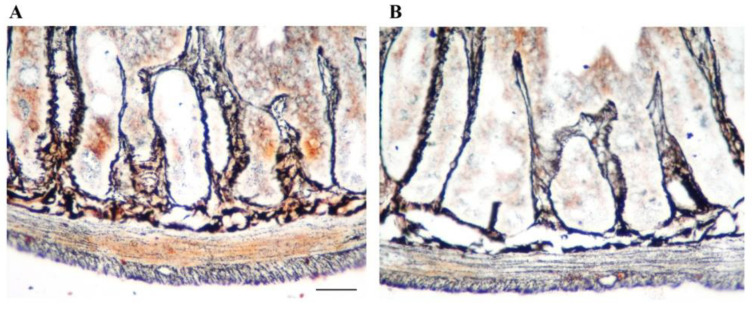
Silver staining on sections of mouse duodenum according to the Bilshovsky method modified by Sukhanov and Vakulin (2002) [34]: after deparaffinization of paraffin sections glued to the slides, they are repeatedly fixed in warm formalin and then treated with silver nitrate with heating. The neuronal fibrillar system is evidenced in control animals (**A**). The staining is less evident and interrupted in animals treated with the parkinsonian neurotoxin MPTP (**B**). Densitometric analysis showed a reduction in silver staining of about 30% following MPTP. These unpublished preliminary data were obtained in animals treated according to the experimental protocol previously published [11]. Scale bar = 70 μm.

**Figure 3:**
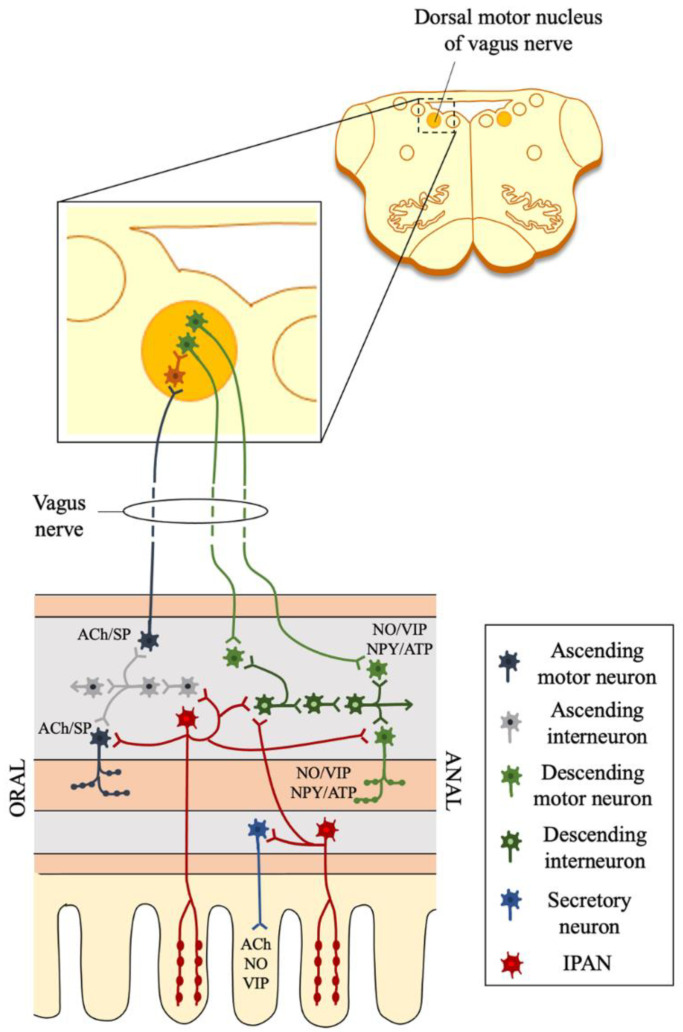
Autonomic parasympathetic input from the dorsal motor nucleus of the vagus nerve connecting the CNS with the ENS. The cartoon summarizes the main types of ENS neurons, including intrinsic primary afferent neurons (IPANs), ascending (orally directed) and descending (anally directed) interneurons, secretomotor neurons, and inhibitory and excitatory motor neurons. IPANs, which are located in the submucosal and myenteric plexuses, project both orally and anally to make synapses with all ENS cell types.

It is now well known that intestinal nerves include extrinsic and intrinsic components. The extrinsic component consists of the classic parasympathetic and sympathetic divisions of the neurovegetative system, while the intrinsic one, that is, the ENS, contains a great number of neurons, whose cell bodies lie within the gut wall (Figure 2A), as well as axonal endings of extrinsic efferents and afferents sympathetic and parasympathetic neurons, whose cell bodies lie outside the gut (Figure 3). Finally, enteric glial cells are also important cytotypes within the ENS. All these components are organized into a complex network for a semiautonomous regulation of GI activities. Then, the ENS controls GI functions independently of extrinsic parasympathetic and sympathetic innervations, although these extrinsic nerve terminals modulate and regulate the ENS [1,3,30,31].

A large variety of neurons and neurotransmitters are present in the ENS, hence the definition of “second brain”. These neurons are organized into ganglionated (clusters of neuronal cell bodies) or non-ganglionated plexuses (nerve fiber strands commonly containing only axons and glial cells), with differences across intestinal regions and among species. Neuronal heterogeneity also depends on sex, age, circadian phase, and disease. The principal ganglionated plexuses are represented by the Auerbach’s myenteric plexus, lying between the inner circular and outer longitudinal muscular layers, and two submucosal plexuses: the outer submucosal plexus (Schabadasch’s plexus), situated adjacent to the circular muscular layer, and the inner submucosal plexus (Meissner’s plexus), which is close to the *muscularis mucosae*. At present, in humans, about 20 functional classes of neurons have been identified [1,32,33].

### 3.1. ENS Cell Types

Intrinsic neurons of the ENS can be classified according to different criteria and approaches: morphological features (neuron size and distribution, and axonal projections, by means of light and electron microscopy), electrophysiological properties, and biochemical markers (neurotransmitter content and receptors, by means of histochemistry, immunohistochemistry, and transcript analysis). These investigations, combined with electrophysiological and nerve lesion techniques, can be useful in defining the circuitry of intrinsic enteric neurons [30,35,36].

From a morphological point of view, a comprehensive classification of cells belonging to the ENS is still lacking. Many studies focused their attention on classifying enteric neurons, and the current knowledge is largely derived from experimental studies in laboratory animals, in particular, guinea pigs. The availability of surgical or bioptic specimens of the human GI tract, particularly from the large intestine, allows studying human tissue directly, as well.

Several types of intrinsic neurons that belong to the ENS have been described (Figure 3). These include intrinsic primary afferent neurons (IPANs) possessing chemo- and mechano-sensory properties, interneurons (one type of orally directed ascending and three types of anally directed descending interneurons), efferent neurons regulating endocrine cells, inhibitory and excitatory motor neurons, and secretomotor and vasomotor neurons [3,36,37,38].

Although several different morphologic types of neurons have been identified in the ENS, enteric neurons with the most correspondent morphology between humans and rodents are classified into two principal types: Dogiel type I neurons have many club-shaped processes and a single long, slender process, whereas Dogiel type II neurons are multipolar and have many long, smooth processes. Type I includes motor neurons consisting of two distinct classes: nitrergic inhibitory and excitatory cholinergic neurons. Each subtype encompasses approximately 5–10% of all enteric neurons within the myenteric plexus. IPANs are thought to be identified with Dogiel type II morphology, with a large, smooth cell body, no dendrites, and several long, uniformly branching axons. They account for approximately 10% of all human myenteric neurons. Other classes of neurons are less consistent across species [3,33,39,40].

Apart from the pioneering Dogiel’s classification, several attempts have been made to obtain a more accurate morphological description of enteric neurons in different species, including humans, and additional cell populations were reported. In particular, refined morphological classifications have been proposed mainly in two species, the pig and the guinea pig. Nevertheless, it remains very difficult to have complete and reliable interspecies comparisons. In any case, basic morphological neuron types can be associated with a general division into three major GI functional groups. (1) Intrinsic primary afferent neurons (these sensory cells are the first link of neuronal reflex arcs and can be activated by chemical and/or mechanical stimuli); (2) different types of interneurons projecting anally, orally, or within the same segment; (3) different types of effector neurons innervating the muscle layers of both gut wall and blood vessels, the mucosa, and likely immune cells. This approach is valid throughout species and accounts for the corresponding roles of different neurons within their respective enteric circuits. Most of the orally projecting circular muscle motor neurons contained tachykinins (TKs) and choline acetyltransferase (ChAT), whereas most anally directed muscle motor neurons resulted positive for vasoactive intestinal peptide (VIP) and neuronal nitric oxide synthase (nNOS) and were partly co-reactive for both markers [33,39].

From an electrophysiological point of view, two major classes of myenteric neurons have been classically distinguished in guinea pigs: S (synaptic) neurons, receiving fast excitatory synaptic input, and AH (after-hyperpolarization) neurons, exhibiting a prolonged after-hyperpolarization. They morphologically correspond to Dogiel type I and II, respectively [39,41,42]. A method to combine intracellular recording with simultaneous morphological identification of neurons in the intact myenteric plexus of human colon ex vivo was developed with the use of 5,6-carboxyfluorescein, a fluorescent dye in microelectrodes that allows recordings from cells in situ. With this approach, it was possible to pair the morphology of every recorded cell with its electrical property. Accordingly, the majority of the myenteric neurons were identified as Dogiel type I neurons with S type electrophysiology. Two neurons had Dogiel type II morphology, and one had a filamentous soma-dendritic morphology. A slow after-hyperpolarization was evoked only in one of the two Dogiel type II neurons. Then, in humans, an apparent scarcity of AH neurons seems to occur [43].

Other studies characterized ENS subpopulations using mRNA sequencing and collectively identified more than 20 enteric neuron cytotypes, with 8–14 myenteric neuron subtypes and 4–7 types of enteric glial cells. These novel approaches allowed us to investigate cells that cannot be readily dissociated and bring useful insight into transcriptomics of the adult mouse and human ENS to identify distinct populations of ENS cells that vary according to species and location along the GI tract [44,45].

More recently, Drokhlyansky et al. [46] developed two methods to enable single-nucleus RNA-seq (snRNA-seq) of human and mouse ENS cells: ribosomes and intact single-nucleus sequencing (RAISIN-seq) RNA-seq for isolation of intact nuclei and ribosome-bound RNA, and mining rare cells sequencing (MIRACL-seq) for label-free profiling of rare cell types. Neuronal and glial cell subsets have been identified by the expression of classic neurotransmitters and markers. In the mouse, ENS neurons were grouped into two major divisions, including either cholinergic or nitrergic subsets, which were correlated with other genes. For example, expression of glial cell line-derived neurotrophic factor (GDNF) family receptors α1 (*Gfra1*) and α2 (*Gfra2*) segregate nitrergic *Nos1* and cholinergic Chat-expressing neurons, respectively. *Gfra1* and *Gfra2* are co-receptors for the GDNF receptor, Ret, which is necessary for ENS formation. Again, *Chat-* and *Nos1*-expressing subsets also differentially showed the transcription factors *Casz1* and *Etv1*. Based on the expression of known marker genes, 21 cell subsets were recognized and subdivided into five major groups:

(1) Five subsets of *Chat+Tac1*+ putative excitatory motor neurons.
(2) Seven subsets of *Nos1+* putative inhibitory motor neurons (four subsets are *Nos1*+*Vip*+), which together coordinate muscle contraction and relaxation.
(3) Four subsets of calcitonin gene-related peptide (*CGRP+*) producing putative sensory neurons, which sense and respond to chemical and mechanical stimuli. These neurons can be largely distinguished by the expression of distinct sensory and effector genes. For example, a subset expressed cholecystokinin (*Cck*) and vasoactive intestinal polypeptide (*Vip*), markers of intestinofugal neurons, or brain-derived neurotrophic factor (*Bdnf*), which is increased in patients affected by irritable bowel syndrome, or Piezo2, a mechanosensitive ion channel involved in the regulation of smooth muscle tone. A second subset was strongly enriched for somatostatin (*Sst*). Another subset expressed Noggin (*Nog*), a bone morphogenetic protein antagonist important to maintain the intestinal stem cell niche, and Neuromedin U (*Nmu*), which activates innate lymphoid cells.
(4) Three subsets of *Penk*+ putative interneurons, which relay signals between neurons. Furness [36] listed six subtypes of interneurons: (a) descending interneurons that signal via ACh, serotonin, and ATP; (b) descending *Nos1+Vip+Grp+Chat*- interneurons; (c) descending *Vip+Chat+Nos1*+ interneurons with ATP signaling; (d) descending Chat+*Sst*+ interneurons; (e) descending *Penk*+ interneurons (responsive to Sst); (f) ascending *Chat+Penk*+ interneurons with ATP signaling.
(5) Two subsets of *Glp2r*+ putative secretomotor/vasodilator neurons, including *Vip*+ non-cholinergic and *Chat*+ cholinergic subsets, which trigger secretions and fluid movement in other cell types. The latter also expressed galanin (*Gal*), as in neurons that innervate the epithelium and arterioles, neuropeptide Y, as in secretomotor neurons, and glutamate decarboxylase 2 (*Gad2*), possibly forming cholinergic/GABAergic neurons [46].

Each neuron subset also expresses unique marker genes, including D2 dopamine receptor, adrenomedullin, prolactin receptor, melatonin receptor, follistatin, Lgr5, and Csf2 receptor. Interestingly, putative motor neurons expressed the mechanosensitive ion channel *Piezo1*, suggesting that they may directly sense distention. Unlike the wide transcriptional heterogeneity of neurons, glial cells clustered into three subsets, each characterized by receptors and transporters. For example, Glia1 was enriched for the GDNF receptor *Gfra2*, Glia2 for the monoamine transporter *Slc18a2*, and Glia3 for the neurotensin receptor *Ntsr1* [46].

In humans, 1445 enteric neurons were sequenced and finally clustered into 14 subsets (4 putative excitatory motor neurons, 5 putative inhibitory motor neurons, 2 putative interneurons, 1 putative sensory neuron, and 2 putative secretomotor/vasodilator neurons subsets), whereas glial cells include 3 subsets. Human ENS subsets share core transcriptional programs with the mouse [46].

In a single-cell transcriptome analysis of the developing ENS, evidence was provided for a novel taxonomy of 12 myenteric neuron classes of the mouse small intestine, taking into account their embryonic diversification. Then, considering that ENS progenitor cells are migratory and can lack distinct positional identities, a new model of enteric neuron specification was proposed, with only two subsets of enteric neuron subclasses (ENC1 and ENC8), which are generated as dividing progenitor cells during neurogenesis. Subsequent identity conversions of immature neurons occur at the postmitotic state when the initial features are down-regulated and replaced by other transcriptional programs, with the formation of the other classes (ENC2-7 and ENC9-12). Thus, the 12 ENCs were generated from an initial binary difference. It can be concluded that the developing ENS is characterized by flexible postnatal neurons. This might explain how neuronal complexity can be formed without spatially defined stem cell populations [47].

A transcriptional profile of genes was carried out in both human and mouse enteric neurons, which were examined from myenteric ganglia and adjacent smooth muscle isolated by laser-capture microdissection. Nuclei from myenteric neurons were taken and subjected to snRNA sequencing. Several enteric neuron subtypes in the duodenum, ileum, and colon were conserved between humans and mice, but multiple species differences were also observed. So, the examination of different regions of the GI tract with snRNA-Seq allowed us to identify 22 myenteric neuron subtypes throughout the entire intestine, and evidence was provided that multiple neuron subtypes have distinct, regional expression patterns of genes in the gut of both humans and mice [40].

In another recent article, additional RNA-seq data from the human and murine ENS were provided. The gene expression of ENS cell subtypes was investigated by means of snRNA-seq on adult mouse and human colon myenteric plexus and single-cell RNA-seq on E17.5 mouse. This study demonstrated new roles for GDNF and neurturin (NRTN), which are two structurally related, potent neurotrophic factors involved in the control of neuron survival and differentiation. *Gfra* genes encode for GDNF-family receptor-α (GFRA) proteins, which are a group of co-receptors forming complexes with GDNF-family ligands. It was found that nitrergic neurons expressed *Gfra1*, while cholinergic neurons expressed *Gfra2* in adult and E17.5 mouse myenteric plexuses. Since GFRA1 and GFRA2 are cell surface receptors that bind preferentially to GDNF and NRTN, respectively, it was shown the occurrence of different effects of these trophic factors on the calcium indicator GCaMP6s activity in enteric neurons, with procontractility effects of GDNF. It was also found that *Tbx3*, which encodes for T-box transcription factor (TBX3), was preferentially expressed in neuronal nitric oxide synthase (NOS1)-producing myenteric neurons, and mice lacking *Tbx3* in the ENS had a 30% reduction in NOS1 neurons [45].

Different types of neurons in the myenteric plexus of human colon were also characterized using retrograde tracing combined with multiple labeling immunohistochemistry. The fluorescent carbocyanine tracer 1,1′-didodecyl-3,3,3′,3′-tetramethylindocarbocyanine perchlorate (DiI) was applied to the myenteric plexus in ex vivo preparations, filling neurons projecting within the plexus. Long ascending and descending putative interneurons were then identified by labeling for different immunohistochemical markers: calbindin (Calb), calretinin, enkephalin (ENK), 5-HT, nNOS, and substance P (SP). In particular, the results showed that calretinin was expressed in very few circular muscle motor neurons, whereas calbindin was detected in 15–20% of both ascending and descending motor neurons. The data showed that in the myenteric plexus of human large intestine, there are four classes of ascending interneurons (ChAT+/ENK+/SP+; ChAT+/ENK+; ChAT+/ENK+/Calb+; ChAT+/Calb+) and four classes of long descending interneurons (ChAT+/NOS+; ChAT+/Calret+; ChAT+/5HT+ cells with or without NOS; NOS+ cells without ChAT). It can be noted that all long ascending neurons were immunoreactive for ChAT, whereas only some long descending neurons were cholinergic. Long descending projections run for up to 70 mm down the colon, whereas long ascending neurons project for up to 43 mm. These long descending and ascending myenteric neurons would act as putative interneurons in polysynaptic polarized reflex pathways involved in propulsion and other possible intestinal functions [48].

Considering the neuronal variety of the ENS population, it is not surprising to find a complex neurochemical transmission within this network. More than 50 neurotransmitters with different biochemical nature have been identified. In fact, apart from acetylcholine (ACh) and noradrenaline (NE), many other neurotransmitters can be released or co-released in the ENS: dopamine (DA), serotonin (5-HT), gamma-aminobutyric acid (GABA), glutamate, nitric oxide (NO), and several neuropeptides, including VIP, neuropeptide Y (NPY), calcitonin gene-related peptide (CGRP), galanin, motilin, adenosine triphosphate (ATP), TK, neurokinin (NK), endogenous cannabinoids and opioids, SP, gastrin-releasing peptide (GRP), somatostatin, and cholecystokinin (CCK) [36,38]. It is noteworthy to note that, in spite of this biochemical richness, the only local catecholaminergic neurotransmitter in the ENS seems to be DA [3,49]. Furthermore, some of these neurochemical agents have not been demonstrated to behave as neurotransmitters. This is the case of glutamate, whose activity in the ENS is weak or absent. Finally, the important role attributed in recent years to mucosal endogenous 5-HT for GI motility is now questioned. In fact, several experimental pieces of evidence showed that pharmacological or genetic impairment of mucosal 5-HT synthesis does not reduce GI transit in vivo. This neurotransmitter/entero-hormone, together with CCK, modulates ENS activity and GI motor patterns. This occurs through the activation of chemosensory stimulants applied to the lumen, along with segmental contractions. When released from enterochromaffin cells, 5-HT activates intrinsic and extrinsic sensory terminals in the lamina propria, and its action on IPANs induces propulsive contractions involving a tachykinergic intermediate [42,49]. Although histamine is not produced by intrinsic or extrinsic GI neurons but mainly released by mast cells and enterochromaffin-like cells, it can act on enteric neuronal circuits and extrinsic afferents, especially in pathological conditions [49].

Another important cell population in the ENS is represented by glia. Like enteric neurons, it derives from the vagal and sacral neural crest. Enteric glial cells are present in ganglia as well as extraganglionic sites and are different from other peripheral glial cells, resembling astrocytes of the CNS. They outnumber neurons, displaying cell-to-cell coupling, and have trophic and protective functions for neurons. Apart from a mere supportive activity, these special cells are also involved in neurotransmission and may represent a link between the nervous and the immune system. Then, loss or functional impairment of glial cells is related to gut diseases. Most typical enteric glial cells are immunoreactive for S-100 or glial fibrillary acidic protein (GFAP) [33].

Several data suggested structural and chemical heterogeneity among enteric glial cells. In the GI tract of adult mice, four morphologically distinct subpopulations of glial cells have been distinguished using Mosaic Analysis with Double Markers and Cre/LoxP technology, carrying out in vivo fate mapping and inducible lineage tracing. Data showed that the morphologically distinct subsets of enteric glial cells expressed unique combinations of glial markers in response to signals from their microenvironment and/or as a result of functional specialization. This suggests that all subtypes of enteric glial cells originate from a common progenitor, which generates different subtypes, depending on its final location and physiological context. This ability to acquire different properties is not restricted to embryonic or early postnatal stages but is maintained throughout adult life, indicating the occurrence of a certain degree of phenotypic plasticity [50].

**Figure 4:**
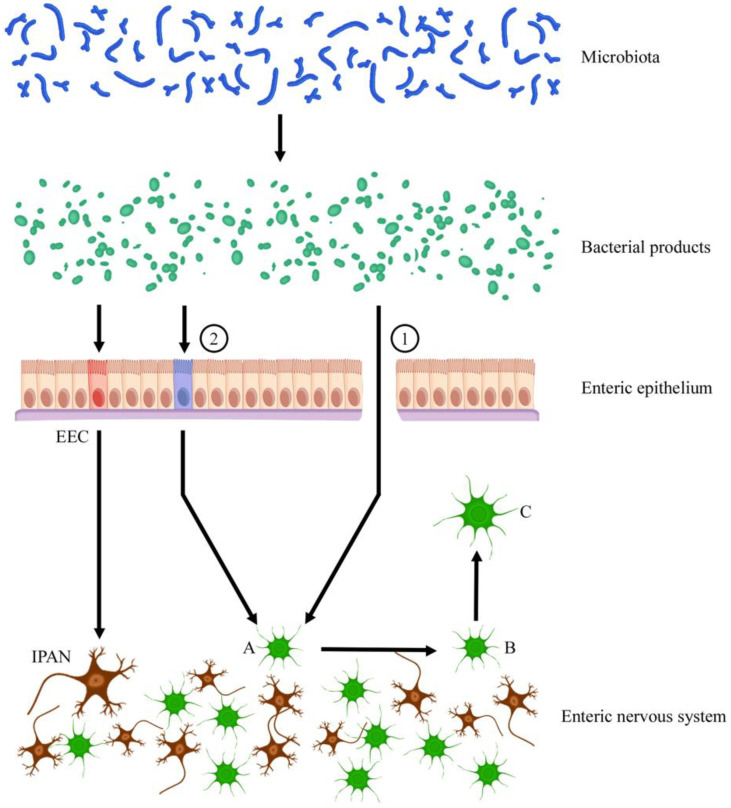
Role of microbiota in the migration of glial cells from the enteric plexus (neurons in brown and glial cells in green) to the lamina propria of the gut mucosa. Bacterial products, such as short-chain fatty acids, either directly (1); through epithelial leakage) or indirectly (2); through recognition of epithelial or immune cells; blue cell), promote the transition of mature quiescent glial cells (**A**) within the intestinal plexi to an active state (**B**) leading to mucosal enteric glial cell progeny (**C**) that migrates into the mucosa. Nerve endings of intrinsic primary afferent neurons (IPANs) do not have direct contact with the luminal content, and specialized entero-endocrine cells (EECs; red cell) scattered throughout the intestinal epithelium provide for sensing the luminal milieu and then communicating these signals to the ENS, as well as to release hormones via endocrine or paracrine mechanisms. Furthermore, IPANs can also detect bacterial products released by microbiota.

Remarkably, enteric glial cells residing in the intestinal lamina propria develop after birth and correlates with the maturation of the gut microbiota. This glial network renews continuously throughout life by incorporating new cells coming from other areas of the gut wall in the process of directional movement, which is controlled by the lumen gut microbiota. Then, the microbiota are able to drive the migration of glial cells into the lamina propria. It was speculated that glial cells may respond to bacterial products, such as short-chain fatty acids, in particular butyric acid. Alternatively, bacterial products may induce intestinal epithelial cells or immune cells to release chemotactic mediators [51] (Figure 4).

Excitatory or inhibitory neurotransmission modulates motor activity within ENS through another important cell type: interstitial cells of Cajal (ICCs). Although they are not neurons, similar to what occurs in the heart, these cells act as pacemakers driving gut motor function, being also sensitive to mechanical activity. They likely operate by means of gap junctions and generate electrical oscillations with slow waves [33]. In both the small and large intestine, ICCs have been observed within the circular and longitudinal muscle layers, in the interlamellar connective tissue of the circular muscle, and in the submuscular plexus, at the submucosal-circular muscle border [52].

Significantly, another cell type shares the same localization of ICCs, which own a specific phenotype: negative for c-kit (CD117) and S100, and positive for CD34 and platelet-derived growth factor receptor α (PDGFRα). About 10 years ago, these cells were named telocytes by Popescu and Faussone-Pellegrini [53]. They are characterized by the presence of long, thin cytoplasmic projections called telopodes. These cells have also been localized in several other organs. In the human ileum and colon, telocytes form with their telopodes an almost complete sheath around both myenteric and submucosal ganglia and interganglionic fascicles. ICCs and telocytes were found differently arranged in enteric plexuses [52].

### 3.2. ENS Circuits and Functions

Apart from the mere description of the neuronal network in the ENS, the functional coordination of this large neuron population remains to be elucidated. Moreover, some studies suggested that there is a distinct clustering of clonally related neurons in enteric circuits, with the formation of overlapping clonal units and the generation of synchronous activity following local stimulation of internodal strands. In this way, large populations of enteric neurons can be synaptically activated at the same time. Finally, this polarized neural circuit is able to involve the convergence and synaptic recruitment of ascending and descending interneurons in order to coordinate the progression of the intestinal content [42].

The modern wireless optogenetics technique, based on the use of different wavelengths of light to recruit light-sensitive ion channels, was adopted to selectively stimulate or inhibit particular neurochemical classes of neurons also in the ENS, allowing the investigation of neural circuits. Then, for example, this new method can be used to stimulate the ENS cell population and increase colonic transit in conscious, freely moving animals. Finally, it can be hypothesized to control gut motility and transit in conscious non-transgenic animals using wireless optogenetics after injection of adeno-associated viruses into the enteric wall [42].

As far as the enteric motor activity is concerned, it is clear that excitatory and inhibitory motor neurons are polarized, with ascending interneurons coupled to excitatory motor neurons and descending interneurons coupled to inhibitory motor neurons. Then, monosynaptic reflexes would induce oral contractions and anal dilatations. Anyway, a localized activity can arise as a consequence of an imbalance of this mechanism. This switch would produce an alternation of peristalsis and segmentation. It was shown that cholinergic and nitrergic motor neurons are involved in a different manner in the generation of the rhythmic propulsive contractions due to migrating motor complexes. Not surprisingly, enteric motor neurons innervate ICCs, but the role of this crosstalk needs to be clarified. Finally, the general arrangement of the enteric nervous circuits is difficult to be examined, because different GI tracts have different patterns of connectivity [39].

Motor neurons are divided into muscular and secretomotor-vasodilators. The former (Dogiel type I) neuron innervates the circular and longitudinal muscles and the *muscularis mucosae*, causing their contraction or relaxation. As previously anticipated, these neurons innervate the circular and longitudinal muscles, have the cell body in the myenteric plexus, and are excitatory (they use ACh and TK and project in the oral sense) or inhibitory (they use NO and VIP and project in the anal sense). The muscular motor neurons generate, following a regional stimulation, coordinated and polarized muscular responses that allow the progression of the intestinal content by inducing contraction in the oral direction and relaxation in the anal direction. The secretomotor-vasodilator neurons, on the other hand, are mainly located in the submucosal ganglia, where they control both the secretion of ions and water through ACh, and the vasodilation of the submucosal arterioles, through VIP. Some of these neurons affect the transport of glucose through the mucous membrane of the small intestine, a process also regulated by vagal-vagal reflexes, whereas others modulate stomach acid secretion [42].

Extrinsic efferent autonomic nerves are not uniformly distributed along the GI tract. Parasympathetic innervation mainly controls the proximal tract, whereas sympathetic innervation is the distal one. Nerve endings of IPANs do not have direct contact with the luminal content. Specialized entero-endocrine cells (EECs) are scattered throughout the intestinal epithelium to sense the luminal milieu. Then, EECs communicate these signals to the ENS and release hormones via endocrine or paracrine mechanisms. In this way, IPANs also seem to detect products released by microbiota (Figure 2). Activation of innate immune pathways by microbe-associated molecular patterns signaling was also considered. Short-chain fatty acids and lipopolysaccharides are thought to be the major candidates for this crosstalk. However, these mechanisms remain still poorly understood. As far as mechanical stimuli are concerned, IPANs do not seem the unique neurons able to respond to such changes. Since Dogiel type I motor neurons also seem to exhibit mechanosensitive properties, the general term of “mechanosensitive enteric neurons” was proposed [39].

The nervous control of vasodilator and secretomotor reflexes are extremely important for the physiopathology of the GI tract. The ENS regulates mainly the local blood flow, whereas extrinsic nerves seem more relevant during inflammatory processes. Secretomotor circuits were described in the submucosal plexus of experimental animals, with the release of endogenous purines that activate postsynaptic P2Y receptors on cholinergic and VIPergic sensory neurons. The ENS also participates in the regulation of cell proliferation and differentiation at the intestinal crypt level, and ACh and 5-HT are involved in this fundamental function [39].

The immune system is an extremely complex orchestra deputed to defense, especially against host pathogens and cancer cells. This control is particularly important in the GI tract for the continuous challenge of microbiota. Both extrinsic and intrinsic innervations contribute to regulating the enteric immune systems. Intrinsic submucosal sensory neurons may detect invading pathogens and regulate the follicular blood supply and lymphocyte trafficking [39,49].

Enteric glial cells are likely to represent the GI counterpart of brain astrocytes and take a significant part in ENS activities, participating in the regulation of neuron signaling. In contrast to brain astrocytes, enteric glial cells are readily accessible to biopsy and can be easily analyzed in patients. They are immunoreactive for three canonical glial markers, that is GFAP, S100-beta, and Sox-10 [54,55]. The enteric neuron-to-glia communication is purinergic, and glia cells express different nucleotide receptors, including P2Y1, P2Y4, and A2B receptors. Neuroglia is also involved in the control of motor activity, and this regulatory function is mediated by different subtypes of muscarinic receptors. When glial calcium signaling was abolished in Cx43 knockout mice, neurogenic secretory responses diminished, indicating that enteric glia plays a role in GI secretion, as well [39]. In mice, enteric group 3 innate lymphoid cells (ILC3), expressing the neuroregulatory receptor RET, sense the environment and control the gut defense. Enteric glial cells participate in the process by sensing microenvironmental stimuli and producing neurotrophic GDNF-family ligands (GFLs). These, in turn, act on ILC3s to promote the interleukin-22 release and barrier defense. This interaction allows envisaging a glial-ILC3-epithelial cell unit, which is orchestrated by neurotrophic factors [56].

## 4. ENS and Parkinson’s Disease

Apart from a physiological degenerative process due to aging, which mainly compromises cholinergic enteric neurons [57], ENS can also be affected by inflammatory and neurodegenerative pathologies, which severely impair the quality of life of patients. In particular, it is now well known that besides neurologic manifestations due to central dopaminergic neuronal loss, with severe disruption of motor activity, Parkinson’s disease is often associated with a general autonomic impairment, and the GI tract is extensively affected, with hypersalivation, oro-pharyngeal and esophageal dysphagia, gastroparesis, small intestine, and colonic dysmotility, and anorectal dysfunction. However, constipation is the main symptom that impairs the quality of life of patients. Moreover, these autonomic alterations anticipate the neurological ones [10,12,58].

In light of these clinical manifestations, the attention was focused on the GI tract in an attempt to find morphological alterations that substantiate GI Parkinsonism. In fact, in the past four decades, several studies showed a neurodegenerative process in the GI tract similar to what occurs in the CNS. In particular, both enteric dopaminergic neuronal loss and occurrence of marker for neuronal degeneration in Parkinson’s disease, including α-synuclein and Lewy bodies deposits in enteric cells, were found [13]. So, esophageal or colonic Lewy bodies were found in two Parkinson’s disease patients with dysphagia, but not in patients without dysphagia or in controls [59]. A reduced number of dopaminergic neurons in the colon of constipated Parkinson’s patients was reported in postmortem and bioptic studies, and in some patients, lower levels of dopamine were measured with high-performance liquid chromatography in the muscularis externa. In line with this, tyrosine hydroxylase (TH)-positive enteric dopaminergic neurons gained increased interest to understand GI dysfunctions in Parkinson’s disease. However, other than dopamine, TH also takes part in the synthetic pathway of other active neurotransmitters, including NA and adrenaline. Furthermore, in the GI tract, TH-immunopositivity was found in VIP- rather than dopamine-containing neurons, confirming that the presence of this enzyme does not unequivocally reflect the involvement of dopamine [60]. Similar results were obtained in a study where the occurrence of Lewy bodies was assessed in enteric specimens of autopsied patients. Because TH is an integral part of dopamine synthesis, the assumption was that Lewy’s bodies occur in dopaminergic TH-containing neurons. However, the hallmark of Parkinson’s disease consists in Lewy bodies within VIP-immunoreactive rather than TH-containing neuronal cell bodies neurons [61]. Thus, the putative enteric dopaminergic neurons may not be so altered in Parkinson’s disease gut [49]. In routine colonic biopsies, phosphorylated α-synuclein immunoreactive neuritis was found in the submucosal ganglia of patients [62].

More recently, in jejunum and colon from some patients who died of Parkinson’s disease, simple conventional staining with hematoxylin and eosin was performed, showing the presence of atrophic or pycnotic neurons in both myenteric and submucosal plexuses. Furthermore, degenerative alterations were accompanied by α-synuclein deposits, which were also present in some preserved neurons, suggesting that accumulation of α-synuclein anticipates neurodegeneration [63]. α-Synuclein aggregates are detected in the brain or GI tract from Parkinson’s disease or dementia with Lewy bodies patients. This was detected by protein misfolding cyclic amplification of real-time quaking-induced conversion assays [64]. An abundance of misfolded aggregates of α-synuclein in the appendix has been shown to foster protein spreading, and appendectomy seems to correlate with a reduced risk of developing Parkinson’s disease [65].

In contrast, other studies could not replicate these alterations found in human enteric biopsies. In fact, Corbillé et al. [66] reported no evidence of dopaminergic and noradrenergic neuronal loss in the submucosal plexus in Parkinson’s disease, suggesting that neuropathology in submucosal neurons is unlikely to be a key for GI dysfunction. Again, another study could not document neuronal loss within the myenteric plexus of Parkinson’s disease patients. This was documented by a quantitative assay of NO, VIP, and catecholamine neurons and α-synuclein neuritis. In the same study, Lewy bodies’ pathology was only rarely co-localized with TH [67].

The importance of the GI tract involvement in Parkinson’s disease led to the development of experimental animal models of this pathology. There are some in vivo animal models of Parkinson’s disease, including the administration of Parkinsonism-inducing neurotoxins: 6-hydroxydopamine (6-OHDA) rat model, 1-methyl-4-phenyl-1,2,3,6,-tetrahydropyridine (MPTP) mouse model, and the chronic rotenone exposure in rats. The MPTP model, where the mitochondrial complex I is impaired, appeared suitable and effective [10]. For an overview of animal models of experimental GI disturbances occurring during Parkinson’s disease, see also Pellegrini et al. [68,69] and Harsanyiova et al. [70].

In line with this model, MPTP was administered to C57Bl/6 mice (60 mg/kg). Ten days after treatment, there was a 40% reduction in dopaminergic neurons in the ENS, while no differences in the density of cholinergic or nitric oxide neurons were observed [71].

In a previous experimental study, we also tried to reproduce digestive alterations observed in patients affected by Parkinson’s disease by intraperitoneal administration of MPTP to C57BL mice (20 mg/kg × 3, 2 h apart). More in-depth, TH-immunopositive neurons were severely lost in both myenteric and submucosal plexuses in the small intestine, up to the axes of the villi, but not in the colon, esophagus, and stomach. This was also evidenced by silver staining, with a reduced neuronal fibrillar network in treated animals (Figure 2B). These observations were confirmed by immunohistochemistry and HPLC, showing the significant loss of DA levels, while NE and 5-HT tissue content was unchanged. Dopamine cell loss was associated with increased α-synuclein immunofluorescence in the duodenum. Thus, the counting of cell immunopositivity for dopamine and noradrenaline transporters in both plexuses indicates that the decrease in TH-immunopositive cells in the small intestine is due to a specific depletion of dopaminergic neurons. All these changes are associated with a significantly reduced colonic transit, which mimics constipation occurring in patients affected by Parkinson’s disease [11].

Motility impairment in the GI tract from the rat was found following chronic intraperitoneal administration of rotenone (2 mg/kg; 5 days/week for 6-weeks), which also inhibits the mitochondrial complex I. From a morphological point of view, loss of small neurons and occurrence of aggregate deposits resembling Lewy bodies were observed in the myenteric plexus [72]. In other studies dealing with this model, GI dysfunctions but not neuronal loss were observed [73], and an alteration of microbiota in both small intestine and colon was also reported [74]. More recently, the chronic systemic exposure to a low dose of rotenone (2.5 mg/kg/day) for 4 weeks by subcutaneous implantation of neurotoxin-filled osmotic mini pump confirmed the usefulness of this model even in mice. In fact, a decreased number of dopaminergic neurons in the substantia nigra pars compacta, as well as a reduction in cholinergic neurons in the dorsal motor nucleus of the vagus and the intestinal myenteric plexus, with an accumulation of α-synuclein, was described both within central and peripheral neurons [75].

In another experimental model, the central nigrostriatal dopaminergic denervation obtained in rats by injecting 6-OHDA into the medial forebrain bundle decreased colonic transit rate. Furthermore, impaired electrically evoked neurogenic cholinergic contractions, enhanced carbachol-induced contractions, decreased basal and electrically stimulated ACh release from colonic tissues. Again, decreased choline acetyltransferase immunopositivity in the neuromuscular layer and increased expression of colonic muscarinic M2 and M3 receptors were observed. This demonstrates that dysregulation of colonic excitatory cholinergic neurotransmission would depend on a loss of myenteric neuronal choline acetyltransferase and a decrease in ACh release [76]. Accordingly, mice overexpressing wild-type human α-synuclein under the Thy1 promoter own alterations in colonic myenteric ganglia and defecation anticipating by several months before the loss of striatal dopamine, which provides an anatomical basis for interference with cholinergic neuronal activation [77]. Table 1 summarizes recent studies about ENS degeneration in different GI tracts.

Native α-synuclein is considered to be abundant in the myenteric plexus of young adult rats. Nevertheless, its propensity to misfold and aggregate leads to α-synuclein aggregates, making this protein a candidate marker for neuropathies in aged animals. In this respect, α-synuclein-positive dystrophic axons were found throughout the GI tract of middle-aged and aged rats, which co-localizes with either NO synthase-, calretinin-, calbindin-, or TH-positive swollen neurites. These findings suggest a complex chronological relationship between the onset of degeneration and the accumulation of α-synuclein or other misfolded proteins [78].

Not surprisingly, a large body of evidence shows a role for enteric glial cells in the pathophysiology of inflammatory and neurodegenerative GI disorders, including Parkinson’s disease. In the GI tract of patients affected by Parkinson’s disease, GFAP and Sox-10 glial markers increase and correlate with pro-inflammatory cytokines. This is observed mostly at the disease onset, while it decreases over time. Considering GFAP phosphorylation as critical in CNS disorders, GFAP phosphorylated at serine 13 is reported in colon biopsies from patients. This reactive gliosis seems to be specific for Parkinson’s disease since it was not found in patients affected by progressive supranuclear palsy or multiple system atrophy [54,55].

In recent times, a brain-gut-microbiota axis has also been recognized. Since the level of some neurotransmitters and metabolites is influenced by intestinal microbiota, that is, the “microbial organ-specific nervous system”, dysbiosis has also been implicated in promoting α-synuclein accumulation, but the matter remains still under debate [79,80]. As shown in humans and in animal models, dysbiosis, especially in terms of bacterial overgrowth and increased intestinal permeability, may initiate inflammation in the ENS. This includes vagus nerve terminals and glial cells and associates with α-synuclein accumulation. In detail, disruption of the intestinal epithelial barrier and alterations of gut microbiota can trigger neuroinflammation within enteric glial cells, thus triggering the onset of Parkinson’s disease with α-synuclein aggregation and accumulation. Subsequently, α-synuclein may propagate to the brain in a prion-like manner just via a pathway involving glia to glia interactions [81]. In addition to this, it has been recently found that Triggering Receptors Expressed on Myeloid cells (TREMs), especially TREM-1, play an important role in those inflammatory alterations affecting both the gut and the microbiota–gut–brain axis, which might be relevant in the pathogenesis of neurodegeneration. Again, inflammation due to microbiota dysbiosis and intestinal barrier impairment may be associated with altered expression of these receptors, and altered cell clearing systems, including autophagy and proteasome [82]. A dysregulation of Toll-like receptor signaling is also implicated in the pathogenesis of α-synucleinopathies [83]. Since GI transit time and intestinal microbiota are highly interrelated, both accelerated and delayed transit may result in dysbiosis. It is difficult to establish the primum movens since, in a vicious circle, the small intestinal bacterial overgrowth is due to impaired motility, which is typical of Parkinson’s disease [84]. Finally, probiotics, diet, and nutritional supplements, as well as fecal microbiota transplantation, have been proposed to slow down the clinical progression of Parkinson’s disease [85]. In this respect, the ENS represents a potential therapeutic target for common GI diseases [86].

## 5. Discussion and Conclusions

It is now ascertained that the complex network of the ENS includes several cell types. Nevertheless, at present, a satisfactory classification of enteric neurons is still lacking. On the other hand, the recognition of different cell types would also depend on the various criteria adopted to distinguish the ENS population. From a morphological, electrophysiological, and biochemical point of view, it was shown that different cell clusters can be dissected. The involvement of enteric immune and glial cells and microbiota makes the understanding of the ENS more and more difficult. For these reasons, GI diseases characterized by a disruption of the ENS deserve special attention to dissect the molecular mechanisms of pathogenesis. Then, as far as the argument of this article is concerned, the peculiar features of central and peripheral Parkinson’s disease represent a challenge to understand when and what really occurs in the GI tract of these patients.

Apart from the motor impairment, it is now well known that in patients affected by Parkinson’s disease, there are also several alterations in all non-motor autonomic functions. The involvement of the GI tract is particularly marked and precedes the onset of motor signs. This means that changes occurring in the ENS can subsequently affect the brain via the vagus nerve, where the misfolded α-synuclein moves retrogradely in a prion-like manner, according to Braak’s hypothesis or independently on the vagus nerve. However, the chronological development of the disease is not so obvious, and bidirectional progress along the brain-gut axis cannot be ruled out. Considering that the central damage in Parkinson’s disease is due to reduced dopaminergic neurons, it appears likely to find similar changes in the GI tract. As a matter of fact, several studies indicate that dopamine is reduced in the gut of patients or in experimental models of Parkinsonism. Nonetheless, these findings were not confirmed in other studies, and changes in cholinergic or VIPergic neurotransmission were also reported since more than one neurotransmitter is altered. The involvement of enteric glial cells is also observed, and this may account for changes in various enteric neuronal types. Finally, the recently discovered prominent role, which is played by microbiota in the GI physiopathology, adds novel insights in our understanding of the intriguing relationships between bacteria, ENS, and glial, endocrine, and immune cells.

In particular, it is now ascertained that several products released by intestinal bacteria, including short-chain fatty acids (SCFAs), can influence the local gut microenvironment, and they are thought to play a pivotal role in microbiota–gut–brain crosstalk”. These bacterial metabolites, such as acetate, propionate, and butyrate, are able to influence several peripheral organs as well as the brain. Moreover, these products were shown to drive the maturation of both glial and neuronal cells. According to this evidence, gut microbiota dysbiosis has been implicated in severe neurodegenerative pathologies, such as Alzheimer’s and Parkinson’s diseases. More in-depth, in Parkinson’s disease patients, it was observed that dysbiosis was associated with reduced production of SCFAs and subsequent increase in endotoxin and neurotoxin production. In line with these findings, growing evidence was provided that fecal microbiota transplantation from healthy donors as well as butyrate administration in animal models of Parkinson’s disease can ameliorate motor symptoms [19,87].

## Figures and Tables

**Table 1:** ENS degeneration in different GI tracts.

Authors (Year)	GI Tract	Species	Parkinson’s Disease Model
Braak et al., 2003 [9]	Distal esophagus, stomach	Human	-
Natale et al., 2010 [11]	Duodenum	Mouse	MPTP
Clairembault et al., 2015 [55]	Descending colon, sigmoid	Human	-
Chung et al., 2021 [58]	Stomach, small intestine	Human	-
Lebouvier et al., 2008 [62]	Colon	Human	-
Ohlsson et al., 2019 [63]	Colon	Human	-
Fenyi et al., 2021 [64]	Stomach	Human	-
Killinger et al., 2019 [65]	Appendix	Human	-
Annerino et al., 2012 [67]	Stomach, duodenum, ileum, colon	Human	-
Anderson et al., 2007 [71]	Colon	Mouse	MPTP
Drolet et al., 2009 [72]	Small intestine	Rat	Rotenone
Miyazaki et al., 2020 [75]	Intestine	Mouse	Rotenone
Fornai et al., 2016 [76]	Colon	Rat	6-OHDA

## Data Availability

Not applicable.
